# Openness to Experience Moderates the Association of Warmth Profiles and Subjective Well-Being in Left-Behind and Non-Left-Behind Youth

**DOI:** 10.3390/ijerph19074103

**Published:** 2022-03-30

**Authors:** Yongfeng Ma, Chunhua Ma, Xiaoyu Lan

**Affiliations:** 1 College of Educational Science and Technology, Northwest Minzu University, Lanzhou 730030, China; mayongfeng@xbmu.edu.cn (Y.M.); mch@xbmu.edu.cn (C.M.); 2 Promenta Research Center, Department of Psychology, University of Oslo, 0373 Oslo, Norway

**Keywords:** subjective well-being, parental warmth, teacher warmth, openness to experience, left-behind youth

## Abstract

Crouched in the socioecological framework, the present research compared the subjective well-being of left-behind youth with their non-left-behind peers. Furthermore, this research investigated the association of parental warmth and teacher warmth using a person-centered approach with adolescents’ subjective well-being on the whole sample, and examined its conditional processes by ascertaining the moderating role of openness to experience and left-behind status in this association. A total of 246 left-behind youth (53.6% girls; *M_age_* = 15.77; *SD* = 1.50) and 492 socio-demographically matched, non-left-behind peers (55.1% girls; *M_age_* = 15.91; *SD* = 1.43) was involved in this study. During school hours, these adolescents were uniformly instructed to complete a set of self-report questionnaires. The results from ANCOVA exhibited no significant differences in subjective well-being between these two groups of youth. Moreover, four warmth profiles were revealed: congruent low, congruent highest, congruent lowest, and incongruent moderate, and youth within the congruent highest profile were more likely than the other three profiles to report higher subjective well-being. Additionally, moderation analyses demonstrated that high openness was one protective factor for subjective well-being, when left-behind youth perceived the lowest levels of parental warmth and teacher warmth congruently. These findings indicate that left-behind youth may not be psychologically disadvantaged in terms of positive psychosocial outcomes, such as subjective well-being, and school activities or social initiatives emphasizing openness to experience would be essential for them to facilitate positive adaptive patterns after parental migration.

## 1. Introduction

With the accelerating industrialization process and dramatic economic growth globally, an unprecedented wave of labor migration has occurred in a few low- or middle-income yet fast-developing countries from South and East Asia, Africa, and Eastern Europe [1,2,3]. Individuals who lived in impoverished regions internationally or internally migrated to developed areas, chasing financially promising job opportunities and better quality of life [4,5,6]. Nevertheless, due to financial considerations, many migrated parents have to leave their children in original communities [7,8]. A plethora of empirical studies have consistently shown that these left-behind youth who are taken care of by their extended family members exhibit high emotional and behavioral difficulties due, mainly, to diminished parental involvement after migration [1,9]. Therefore, examining the correlates of left-behind youth’s mental health would be very important for developing much-needed, evidence-based intervention or prevention programs.

In this study, we leveraged Chinese youth as a reference point in the hope of providing essential insights into worldwide migrant populations and their families. Instead of focusing on a dominant deficit approach, the current study focused on youths’ strengths and subjective well-being (SWB), in line with the positive psychology movement [10]. Specifically, SWB refers to an individual’s evaluation of his or her life quality, encompassing three key aspects: life satisfaction (cognitive aspect), and positive and negative affect (emotional aspect) [11,12]. Applying a socio-ecological framework in the present research [13], we aimed to investigate how youth’s immediate surroundings (e.g., parents and teachers) and their personal characteristics (e.g., openness to experience) were individually and jointly associated with SWB. In what follows in the introduction, we review each of the study variables and present research questions and tested hypotheses, starting from the presentation of parental warmth and teacher warmth.

### 1.1. Parental Warmth and Teacher Warmth Using a Person-Centered Approach

In this study, we focused particularly on parents and teachers, as they represent two prominent sources of social support in youth’s immediate surroundings and are responsive to youth’s needs, which could be helpful for developing more practical and effective strategies in promoting youth’s SWB [14,15]. Specifically, parental warmth and teacher warmth are defined as the quality of the affectional bond that reflects verbal and non-verbal interactions (e.g., expressions of affection and emotional support) between parents/teachers and youth, in line with acceptance-rejection theory [16,17]. Several empirical studies have pointed out that affectionate and warm parents/teachers–youth interaction plays a prominent role in a youth’s healthy emotional development and better SWB [18,19]. Indeed, immediate surroundings with high responsiveness and warmth significantly influence positive emotional climate with high emotional security and a sense of comfort, contributing to adolescents’ positive social and emotional development [20,21,22].

Despite these shreds of solid evidence, several conceptual and methodological limitations still merit further investigation. First, there is a shortage of studies that simultaneously incorporate father warmth, mother warmth, and teacher warmth and investigate their congruent impacts on adolescents’ SWB [23]. This limitation is particularly salient in the context of Chinese culture, as, within one family, the roles of father and mother in disciplining their children can be complementary or significantly distinctive, such as the patterns of “strict father and kind mother” [24,25]. Additionally, the salient role of teachers in students’ socioemotional functions cannot be ignored, as teachers in each classroom have broad responsibilities for adolescents’ academic and daily life activities [26,27]. To gain a more comprehensive understanding of contextual warmth on adolescents’ SWB, it is valuable and informative to incorporate all these three figures simultaneously in one investigation.

Second, most quantitative studies to date have heavily employed a traditional variable-centered approach to study the association of isolated warmth figures with adolescents’ SWB. Built upon the assumption that the population is homogeneous [28], the variable-centered approach fails to consider that adolescents may simultaneously perceive a distinct degree of warmth from different social agencies, such as parents and teachers, and makes it challenging to have a holistic perspective of contextual warmth based on natural configurations in adolescents’ social spheres [29,30]. Due to these inherently methodological limitations, some scholars argue that research should be augmented with a person-centered approach, assuming that there exist numerous unobserved subgroups within a certain population [24,28,31]. Adopting a person-centered approach would allow researchers to explore naturally occurring sub-groups of adolescents combining the differential degree of perceived father warmth, mother warmth, and teacher warmth, and to discern the associations of these emerging warmth profiles with SWB for specific subgroups of adolescents.

In concert with the socio-ecological framework [13], we also aimed to demonstrate whether personality traits, such as openness to experience, may explain conditional processes of contextual warmth with adolescents’ SWB, as we discussed below.

### 1.2. Openness to Experience

Openness to experience refers to individual differences in the proneness to novelty, tolerance of ambiguity, and openness to new ideas [32,33]. Specifically, adolescents with greater openness tend to appreciate novel ideas, actively seek unfamiliar experiences, and experience positive and negative emotions more intensely [32,34]. Although previous studies have highlighted the critical correlates of personality traits, mainly through extraversion and neuroticism, with resilience and SWB, these studies also indicate that openness is an additional important, yet less studied, variable related to SWB [32,35,36].

Importantly, accumulating research has emphasized the vital role of openness in stress regulation, migrants’ intercultural effectiveness, and their successful sociocultural adaptation [37,38,39,40]. Adolescents with greater openness are willing to try different activities, process novel experiences, and life changes, representing a valuable prerequisite for negotiating life changes after parental migration and demonstrating a successful sociocultural adaptation [37,38,41]. Given that adolescents who score high on openness are more appreciative of and responsive to family changes and accept new experiences [42,43], high open adolescents are likely to respond more positively to parental migration and/or low contextual warmth. Therefore, it is essential to investigate whether openness to experience moderates the association between warmth profiles and SWB in left-behind and non-left-behind youth.

### 1.3. The Current Study

The present study investigated the following research questions (RQ). RQ1: do left-behind and non-left-behind youth differ in SWB? As parental migration and subsequent changes in adolescent living arrangements generate significant stress into their life [44,45], we anticipated that left-behind youth might report a lower level of SWB than their non-left-behind peers.

RQ2: Is it possible to identify distinct warmth profiles based on three salient figures (i.e., fathers, mothers, and teachers) using a mixed sample of left-behind and non-left-behind youth? Due to the scarcity of literature concerning warmth profiles based on these three figures, we did not generate a priori hypothesis about the number of warmth profiles that may emerge. However, according to the previous study [23], we expected that the presence and the absence of father/mother warmth could co-occur in combination with the presence and the absence of teacher warmth, thereby producing distinct combinations of congruent/incongruent warmth profiles across these three figures.

RQ3: Are these emerging warmth profiles linked to SWB? Additionally, are these expected associations moderated by openness to experience and/or left-behind status? Given the notable roles of both parents and teachers in adolescents’ SWB [14,15], we hypothesized that a congruent high warmth profile (compared with other congruent low or incongruent patterns) would be more strongly and positively associated with SWB. Moreover, due to parental migration and subsequently diminished capacity to provide direct and sufficient warmth for left-behind youth, high open adolescents may react more positively to decreasing contextual warmth and/or left-behind experiences, and thus report high levels of SWB [41]. Therefore, we expected that openness to experience might buffer against the detrimental effect of low contextual warmth on SWB, which might be particularly pronounced for left-behind youth than their non-left-behind peers.

By addressing these research questions, the current study significantly contributes to extant research in several innovative ways. First and foremost, in contrast to dominant literature focusing on adverse outcomes, the current study focuses on youths’ strengths and SWB, moving away from pathological labeling and fueling the discussion on a more comprehensive understanding of left-behind youth’s adaption. Second, to complement a traditional variable-centered approach, the current study is among the first using a person-centered approach to simultaneously incorporate three significant figures in youth’s social spheres, documenting the association of distinct warmth profiles with youth’s SWB. Third, the present research contributes to the scientific understanding of the association under investigation by innovatively documenting the moderating role of openness to experience and left-behind status therein. This study, therefore, helps to deepen the theoretical understanding of the interrelated associations between youth’s social spheres and their personal characteristics, and provides tangible evidence on targeted intervention or prevention programs aimed at facilitating positive adaptive patterns for youth with left-behind experiences.

## 2. Methods

### 2.1. Participants and Procedures

The current study was based on a cross-sectional design, which was well-suited to address specific research questions under investigation. Prior to data collection, study protocols and consent forms were ethically reviewed by the responsible research ethics committee, ensuring that this study complies with strict ethical standards included in the Declaration of Helsinki for human rights. School principals and head teachers collaborating with our research team approved this project, and informed consent forms were received from parents and adolescents. During class hours, trained research assistants together with head teachers administrated this investigation, and adolescents were asked to collectively complete a set of self-report questionnaires in simplified Chinese lasting approximately one hour, separated by two sessions. During all research processes, adolescents’ participation in this study was voluntary, and collected data were used solely for scientific purposes.

A total of 2671 Chinese adolescents participated in this investigation, with grade levels ranging from 7th to 11th (see Supplementary Materials Table S1 for more details). Eligibility criteria were as follows: (a) adolescents attended public schools and aged between 13 to 18 years old, and (b) for left-behind youth, one or both of their parents migrated to other cities in China for working continually for at least six months. After applying these criteria, 246 left-behind youth (53.6% girls; *M*_age_ = 15.77; *SD* = 1.50) were identified. Among them, 23.1% of left-behind youth reported that both of their parents migrated to other urban regions for work, and 68.3% and 8.6% of left-behind youth reported that one of their fathers and mothers migrated. The duration of their parental migration ranged from 1 to 10 years (*M* = 5.6 years). Most of their fathers (63.2%) and mothers (61.7%) achieved a secondary school education background.

When comparing two independent groups, unequal sample sizes often affect the robustness of the equal variance assumption in equivalence tests, such as ANOVA, leading to invalid statistical inferences [46]. This is particularly pronounced, as samples become increasingly unequal. To bear this in mind, the current study, therefore, focused on the trade-offs between balanced sample size and sufficient statistical power. Although having precisely equal numbers of participants for each group would be an ideal solution, it is possibly difficult to detect potential effects considering the relatively small sample size in the targeted group (*n* = 246). In this perspective, we intentionally doubled the number of non-left-behind youth, following a few empirical studies comparing left-behind with non-left-behind youth [47,48,49]. Notably, it is feasible to have unequal sample sizes in experimental and developmental studies [50]. It has been suggested that the potential bias caused by this relatively unequal sample size (e.g., up to two times in the control group) can be neglectable if the whole sample size is sufficient [46].

Specifically, based on the sample size of left-behind youth, we randomly extracted 492 (55.1% girls; *M*_age_ = 15.91; *SD* = 1.43) non-left-behind youth from the original non-left-behind data pool. We considered this randomly selected sample representative of the original non-left-behind sample, as these two samples did not differ significantly in salient sociodemographic features (i.e., age, gender, and family socioeconomic status) nor in study variables (see Supplementary Materials Table S2). In addition, we conducted a re-sampling method; that is, we randomly re-derived an approximately 25% non-left-behind sample from the original non-left-behind data pool, and the main results presented remained the same.

Furthermore, we conducted a power analysis to determine whether the sample analyzed could demonstrate sufficient statistical power concerning the analyses planned [51]. It was estimated that a minimum of 128 participants for ANCOVA was needed to detect anticipated medium effect size (Cohen’s *d* = 0.50), whereas a minimum of 218 participants for multiple regression with covariates included was required to detect anticipated medium effect size (*f*^2^ = 0.15). Based on our selected sample of left-behind and non-left-behind youth (*N* = 738), we had above 95% statistical power (α = 0.05, 1 − β = 0.95) to make significant statistical inference. Therefore, we concluded that the sample analyzed would be sufficient to demonstrate our RQs.

### 2.2. Measures

#### 2.2.1. Subjective Well-Being

For assessing positive and negative affect, we used the Affect Balance Scale [52,53]. This scale comprises 14 items, with 8 per positive affect and 6 per negative affect. Sample items are “things were going your way (positive affect),” and “very lonely or remote from other people (negative affect).” Participants reported each item on a 4-point scale from 1 (*never*) to 4 (*always*). The mean score for each facet was calculated, with a higher score representing a higher level of a particular affect.

For assessing life satisfaction, we used the Multidimensional Students’ Life Satisfaction Scale [54,55]. This scale consists of 25 items measuring adolescents’ satisfaction in five important domains: family (5 items), friend (5 items), school (5 items), living environment (5 items), and self (5 items). For example, one item is, “my friends treat me well (on the friend domain).” Adolescents indicated his or her degree of agreement with each item on a 4-point scale from 1 (*totally disagree*) to 4 (*totally agree*). In the present study, these five subscales were significantly interrelated in both left-behind (*r* ranged from 0.36 to 0.56) and non-left-behind youth (*r* ranges from 0.35 to 0.60). Concerning these moderate associations among subscales, an average score across these five domains represented adolescents’ multidimensional life satisfaction [56,57].

The current study focused on an aggregated definition that constitutes SWB as adolescents’ overall evaluation of an affect balance and life satisfaction in different dimensions. Therefore, following the procedure of previous studies [58,59], the total score of SWB was calculated in two steps. First, we standardized the scores of positive affect, negative affect, and life satisfaction in the whole sample. Second, we subtracted negative affect from the sum score of positive affect and life satisfaction, with a high score representing a greater degree of SWB in the current study.

#### 2.2.2. Parental Warmth

For assessing parental warmth, we used the subscales of the Parental Acceptance-Rejection/Control Questionnaire [22]. A total of 16 items were used to measure parental warmth, with 8 per father warmth and 8 per mother warmth. One item example is, “My mother/father lets me know she/he loves me”. A 5-point Likert scale from 1 (*almost never true*) and 5 (*almost always true*) was applied to assess each item. The mean of these items separated by each dimension was calculated as the score of father warmth and mother warmth. A higher score represented higher father warmth and mother warmth.

#### 2.2.3. Teacher Warmth

For assessing teacher warmth, we used one of the subscales of the Children’s Appraisals of Teacher Support [60]. The warmth subscale comprises seven items that measure adolescents’ perception of teacher support, encouragement, and acceptance. One item example is, “My teacher likes me”. A 5-point Likert scale from 1 (*completely disagree*) to 5 (*completely agree*) was used. We calculated the mean score of these items, with higher scores representing higher teacher warmth.

#### 2.2.4. Openness to Experience

For assessing openness to experience, we used one of the subscales of the Big Five Inventory [61,62]. This subscale encompasses ten items, and one example item is, “I see myself as someone who is original, comes up with new ideas”. The items are scored on a 5-point Likert scale with anchors from 1 (*strongly disagree*) to 5 (*strongly agree*). The mean score of the 10 items was calculated, with a higher score indicating greater openness to experience.

#### 2.2.5. Left-Behind Status

Youth were asked, “did your father and/or mother migrate to other cities working continually for a long period (at least six months)”? Those youth who reported parental migration were asked additional items to indicate the duration of parental migration and current residential status. These questions were adapted from Zhao et al. [63].

#### 2.2.6. Confounding Variables

Participants were asked to provide information on their age, gender, parents’ education, parents’ occupations, and family monthly income. Following prior research [64], family SES in this study was represented by a standardized, composite score of parents’ education, parents’ occupations, and family monthly income. These socio-demographic variables were controlled for because prior research has documented the potential association of these variables with SWB [65]. Moreover, adolescents answered a 16-item questionnaire to assess their social desirability due to the concern about response artifact of social desirability to self-report questionnaires [66].

### 2.3. Data Analyses

Data analyses were carried out in SPSS 21.0 [67], Mplus 7.0 [68], and Jamovi 2.3 [69]. The missing values in the current dataset were completely at random [70]. They, therefore, were replaced based on the mean score on each of the measurements. Prior to testing research questions and hypotheses, we calculated means and standard deviations for variables of interest to define the characteristics of the present research, and analyzed bivariate correlations between variables.

In terms of RQ1, we used an Analysis of Covariance (ANCOVA) to compare the SWB in two groups of adolescents. In terms of RQ2, we applied a latent profile analysis to explore warmth profiles. The ideal profile solution was selected based on the comparison of model fit indices across distinctive profile solutions, such as low AIC, BIC, aBIC, a significant likelihood ratio test, as well as high entropy [28]. In terms of RQ3, we applied a linear regression analysis, statistically controlled for age, gender, family SES, and social desirability, to examine study associations. In the case of significant two- or three-way interactions in the liner regression, we leveraged mixed Analysis of Variance (ANOVA), coupled with plotted figures, to interpret these interactive patterns, because there were two categorical variables (i.e., warmth profiles and left-behind status) embedded inside the interaction terms. In this context, left-behind status was regarded as a between-subject factor, and warmth profiles and openness to experience were treated as within-subject factors [71]. To increase the accuracy in parameter estimation, we also presented confidence intervals in terms of unstandardized regression coefficients in the linear regression [72].

## 3. Results

### 3.1. Descriptive Statistics and Correlational Analyses

Descriptive statistics and bivariate correlations among study variables, as well as the reliability coefficients (i.e., Cronbach’s alpha) per each measurement, are presented in Table 1 and Table 2, separated for left-behind and non-left-behind youth.

### 3.2. RQ1: Group Differences in Subjective Well-Being

**Figure 1:**
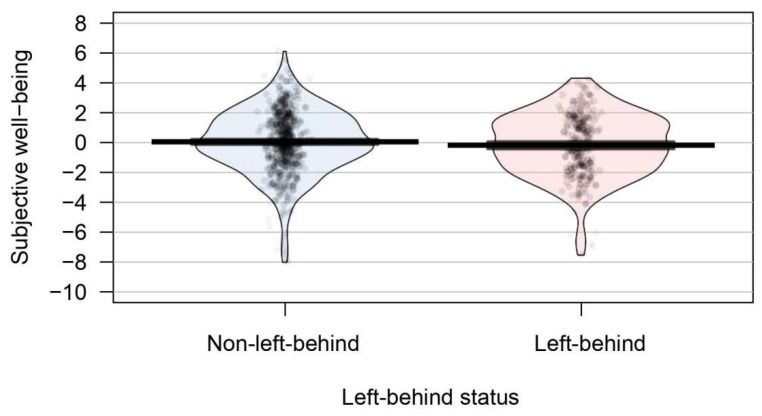
Group differences in subjective well-being. *Note*. *N* = 738. Points—Raw data; Bar/Line—Mean; Bean—Data distribution; Band—Confidence interval.

The mean score of SWB for left-behind youth was −0.14 (*SD* = 2.11) and was 0.07 (*SD* = 2.18) for their non-left-behind peers (see Figure 1). The results based on ANCOVA, after controlling for age, gender, family SES, and social desirability, exhibited that SWB were not significantly different in these two groups of adolescents, *F* (1, 736) = 1.33, *p* = 0.24.

### 3.3. RQ2: Identifying Warmth Profiles

Model fit indices for solutions with one to five latent warmth profiles are presented in Table 3.

As summarized in Table 3, the likelihood ratio tests remained significant for all profile solutions. We first excluded the five-profile solution, as the smallest percentage of this profile only represented less than 5% of the whole sample [73]. Of the remaining profile solutions, the four-profile achieved lower AIC, BIC, adjusted BIC, and almost identical degree of classification accuracy, as demonstrated by entropy values. Therefore, overall speaking, these model fit indices favored the four-profile solution that was employed to analyze subsequently.

**Figure 2:**
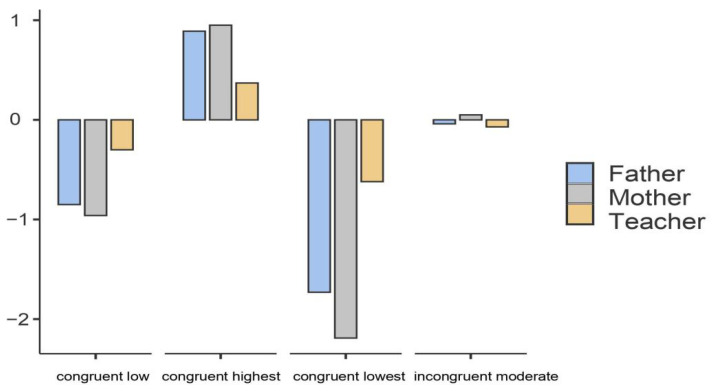
Four warmth profiles based on standardized scores of father warmth, mother warmth, and teacher warmth. *Note*. *N* = 738.

Figure 2 depicts the structure of these emerging four warmth profiles based on standardized scores for each indicator. These profiles were labeled according to Chung et al.’s research [23]. Specifically, youth in the first profile (*n* = 157; 21.3%), named as “congruent low,” reported low scores on father warmth, mother warmth, and teacher warmth; youth in the second profile (*n* = 267; 36.2%), labeled as “congruent highest,” reported the highest scores on father warmth, mother warmth, and teacher warmth; youth in the third profile (*n* = 54; 7.3%), named as “congruent lowest,” displayed the lowest scores on father warmth, mother warmth, and teacher warmth; youth in the fourth profile (*n* = 260; 35.2%), labeled as “incongruent moderate,” showed low-to-moderate scores on father and teacher warmth, but moderate-to-high scores on mother warmth. Mean differences in father warmth, mother warmth, and teacher warmth across these four profiles are represented in the Supplementary Materials Table S3.

### 3.4. RQ3: Associations of Warmth Profiles, Openness to Experience, Left-Behind Status with Subjective Well-Being

Before conducing multiple linear regression, we checked whether the data met each of the assumptions of this analysis, and the corresponding results have been reported in the Supplementary Materials.

Table 4 presents the results of the linear regression analysis, *F* (19, 717) = 22.3, *p* < 0.001, AIC = 2926, BIC = 3022, RMSE = 1.71. In this analysis, we treated the congruent highest profile as the reference group, and compared each of the other three warmth profiles to this group. The model totally explained 37.2% of the variance of SWB.

As shown in Table 4, adolescents within the congruent highest profile were more likely than the other three profiles to report higher SWB. Openness was positively related to SWB. Moreover, a significant three-way interaction (warmth profiles (congruent highest vs. congruent lowest) x openness x left-behind status) was identified.

**Figure 3:**
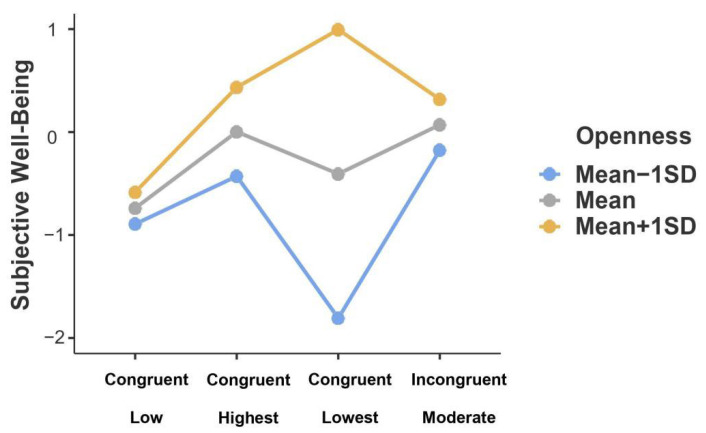
Interaction effect of warmth profiles and openness on subjective well-being in left-behind youth. *Note*. *N* = 246. Openness was divided into three levels based on mean: Mean − 1 SD, Mean, and Mean + 1 SD.

Follow-up post hoc comparisons showed that, in terms of left-behind youth within the congruent lowest profile, those with higher openness reported higher SWB than those with lower openness (*b* = 2.87, *SE* = 0.73, *t* = 3.93, *p*_Bonferroni_ = 0.002). By contrast, for left-behind youth within the congruent highest profile, the scores of SWB were not significantly different across distinct levels of openness (*b* = 0.76, *SE* = 0.40, *t* = 1.91, *p*_Bonferroni_ = 1.00; see Figure 3). It indicated that high openness was a protective factor when left-behind youth perceived the lowest levels of parental and teacher warmth congruently.

**Figure 4:**
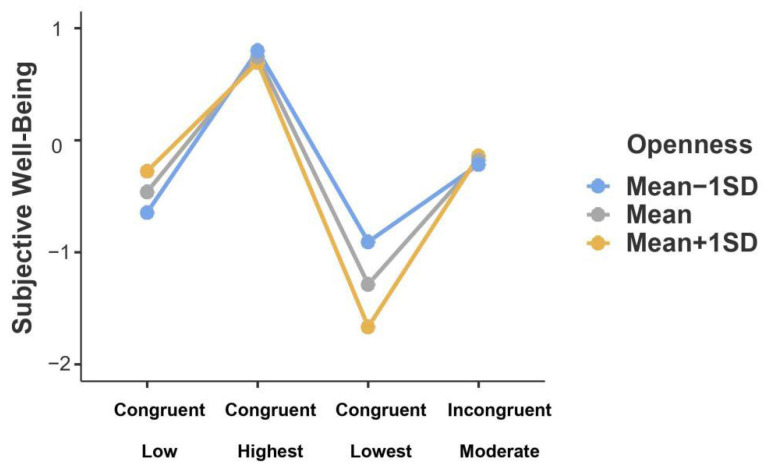
Interaction effect of warmth profiles and openness on subjective well-being in non-left-behind youth. *Note*. *N* = 492. Openness was divided into three levels based on mean: Mean − 1 SD, Mean, and Mean + 1 SD.

With regard to non-left-behind youth within both the congruent lowest profile (*b* = 0.07, *SE* = 0.27, *t* = 0.25, *p*_Bonferroni_ = 1.00) and the congruent highest profile (*b* = 0.23, *SE* = 0.67, *t* = 0.34, *p*_Bonferroni_ = 1.00; see Figure 4), the scores of SWB were not significantly different across distinct levels of openness. Moreover, as shown in Figure 4, youth within the congruent highest profile were more likely to report higher SWB than those within the congruent lowest profile, regardless of the levels of openness (*b* = 2.15, *SE* = 0.53, *t* = 4.05, *p*_Bonferroni_ < 0.001 for higher openness; *b* = 1.99, *SE* = 0.66, *t* = 2.99, *p*_Bonferroni_ = 0.05 for lower openness).

## 4. Discussion

Left-behind youth are rapidly growing and increasingly visible worldwide [1,2,3], but their positive psychological correlates have received little research attention. To narrow these knowledge gaps in the literature, the current research compared the SWB of left-behind and non-left-behind youth. This research, moreover, examined the association of parental warmth and teacher warmth using a person-centered approach with SWB on the combined sample, and investigated whether this association was moderated by openness to experience and left-behind status. Unexpectedly, the findings found no significant differences in SWB across these two groups of adolescents. Moreover, four warmth profiles were revealed: congruent low, congruent highest, congruent lowest, and incongruent moderate. Adolescents within the congruent highest profile were more likely than the other three profiles to report higher SWB. Additionally, moderation analyses exhibited that high openness was regarded as one protective factor for SWB when left-behind youth perceived the lowest levels of parental warmth and teacher warmth congruently.

In terms of RQ1, we compared the SWB of left-behind youth with non-left-behind peers. Contrary to our first hypothesis and the literature concerning SWB of left-behind adolescents in rural China [44,45], the results showed that these two groups of adolescents did not differ in SWB. This finding may indicate that “hopes and hurdles” may coexist in adolescent adaption following parental migration in urban China [74]. One possible explanation could be related to socioeconomic conditions in urban China. Unlike rural areas in contemporary China, urban regions present favorable educational opportunities and socioeconomic conditions [75]. In this context, the possible disadvantage of parental migrant on left-behind adolescents’ SWB may be somehow compensated [64]. Another possible interpretation could be because migration may economically benefit family members left behind, as remittance transfers can ease budget constraints and improve their quality of life. In addition, it is noteworthy that these two groups of adolescents were socio-demographically matched. The similar levels of family SES and related demographic characteristics may potentially weaken the group differences in SWB.

Concerning RQ2, by extending prior research of warmth profiles [23], the current findings revealed four warmth profiles: congruent low, congruent highest, congruent lowest, and incongruent moderate. Of these profiles, the congruent highest represented the largest percentage in the whole sample. This may indicate that parents and teachers are salient figures in adolescents’ social spheres, and provide immense emotional support. However, the measurement per parental warmth and teacher warmth was solely based on self-report questionnaires, and it is possible that adolescents may respond to these items in a socially desirable manner. Thus, this caution should be kept in mind when readers explain this finding. Moreover, the second largest percentage of adolescents fall within the incongruent moderate profile, characterized by moderate-to-high mother warmth yet low-to-moderate father and teacher warmth. One possible explanation for this pattern is that mothers are still regarded as the primary caregiver and prominent figures to offer emotional support in contemporary Chinese society. In addition, the remaining two profiles were characterized by different degrees of low parental and teacher warmth congruently.

Concerning RQ3, we investigated the association of these emerging warmth profiles with SWB, as well as its conditional processes. In line with our expectations, adolescents within the congruent highest profiles are more likely than the other three profiles to report higher levels of SWB. This finding corroborates prior research [18,22,76], suggesting that parents and teachers are significant figures in adolescent daily life and contribute jointly and significantly to their well-being. One possible explanation is that parental warmth and teacher warmth can provide adolescents with high emotional security and a sense of support. These positive experiences accelerate the formation of developmental assets, such as adaptive psychological and behavioral patterns, which enhances adolescents’ SWB [77].

Furthermore, the interactive pattern exhibited that when reporting the lowest levels of parental warmth and teacher warmth congruently, left-behind adolescents with higher openness to experience are more likely to report higher SWB. Adolescents with high openness may tend to effectively regulate life-stress and use adaptive coping strategies in response to parental migration [34,78]. Moreover, as demonstrated by previous studies [79,80], openness is positively related to flexibility and creativity, enabling adolescents to accept or initiate adaptive changes in life. In this regard, left-behind youth with high openness report greater SWB than low-openness adolescents who are more vulnerable to parental migration [39].

When interpreting these significant findings, a few notable limitations of this study should be bear in mind. First, the present research was conducted cross-sectionally, and thus we cannot infer causality concerning the associations under investigation. Second, although self-reported questionnaires employed in the current study have been validated in Chinese adolescents and demonstrated as reasonably appropriate to study these constructs, we cannot entirely exclude the possibility of common method bias that may inflate study associations. Third, our findings should be considered within the cultural boundaries of Chinese society, as the current study was limited by relying on a relatively smaller sample size of Chinese left-behind and non-left-behind youth. Finally, the current study only focused on global SWB, and the in-depth exploration of the domain-specific life satisfaction and emotional aspects would be informative [81].

Despite these limitations, the current study provides substantial theoretical and practical implications. In terms of theory, the current study enriches the socioecological framework among youth with and without left-behind experiences, demonstrating the interactive patterns between their immediate surroundings and personal characteristics. With regard to practical implications, the current study suggests that “hopes and hurdles” may coexist in youth’s psychosocial adaptation after parental migration. Therefore, researchers interested in left-behind youth are recommended to employ both positive and negative outcomes to have a comprehensive understanding of their psychosocial adaption. At the same time, policymakers should implement a few strategies to help reduce “negative stereotypes” on left-behind youth, as they are not always psychologically disadvantaged. Moreover, intervention or prevention strategies should pay specific attention to bolstering support systems and nurturing warm and supportive relationships between youth and their parents/teachers. For instance, practitioners or educators should initiate professional guidance during regular parent–teacher meetings with parents and teachers. They can supervise parents and teachers on how to emotionally express themselves and behaviorally provide warmth in responses to youth’s needs, equipping them with sufficient skills when youth approach them for help. Likewise, practitioners or educators have to highlight the essential role of regular contacts and collaborations between parents and teachers for positive youth development. For school-aged adolescents, their better SWB depends not only on teachers’ supervision and support but also on a congruent format of joint endeavors between parents and teachers. Additionally, left-behind youth are advised to receive some training tasks at school to improve their openness to experience [82,83], ensuring their positive adaption patterns after parental migration. Practitioners or educators should highlight the variety of these designed tasks, incorporate adventurous spirits inside, and gradually bump up the challenges as their skills develop.

## 5. Conclusions

To recap, the findings advance existing scholarship, indicating that left-behind youth may not be disadvantaged in terms of positive psychological outcomes, such as SWB. Perhaps more importantly, for left-behind youth who perceive less contextual warmth from their immediate environments, school activities or social initiatives emphasizing openness to experience would be essential for them to develop positive adaptive patterns after parental migration.

## Figures and Tables

**Table 1:** Descriptive statistics and intercorrelations among variables for left-behind youth.

	M	SD	Range	Alpha	1	2	3	4	5	6	7	8	9	10	11	12
1. Father warmth	3.58	0.95	1–5	0.89	-											
2. Mother warmth	3.83	0.88	1–5	0.87	0.69 ***	-										
3. Teacher warmth	3.73	0.81	1–5	0.88	0.16 *	0.13 *	-									
4. Openness	3.78	0.71	1–5	0.80	0.15 *	0.13 *	0.24 ***	-								
5. Positive affect	2.85	0.35	1–4	0.81	0.10	0.05	0.15 *	0.30 ***	-							
6. Negative affect	2.18	0.38	1–4	0.78	0.10	−0.04	−0.13 *	−0.19 **	−0.46 ***	-						
7. Life satisfaction	3.16	0.47	1–4	0.92	0.56 ***	0.50 ***	0.28 ***	0.39 ***	0.24 ***	−0.09	-					
8. Age	15.77	1.50	13–18	-	0.10	0.13 *	0.05	0.11	0.11	−0.13 *	0.09	-				
9. Gender ^a^	-	-	1–2	-	0.08	0.01	0.02	0.02	0.10	−0.17 **	0.11	0.07	-			
10. Socioeconomic status	16.18	1.96	8–23	-	0.11	0.07	−0.12	0.02	0.01	−0.06	0.05	0.05	0.09	-		
11. Years	6.99	3.90	1–17	-	−0.04	0.01	−0.03	−0.01	0.09	−0.06	−0.03	0.23 ***	0.12	0.06	-	
12. Social desirability	5.25	0.82	1–7	0.85	0.45 ***	0.33 ***	0.29 ***	0.50 ***	0.31 ***	−0.17 **	0.70 ***	0.09	0.12	0.09	−0.02	-

*Note*. *N* = 246. ^a^ coded as 1 = boys and 2 = girls. * *p* < 0.05, ** *p* < 0.01, *** *p* < 0.001.

**Table 2:** Descriptive statistics and intercorrelations among variables for non-left-behind youth.

	M	SD	Range	Alpha	1	2	3	4	5	6	7	8	9	10	11
1. Father warmth	3.75	0.87	1–5	0.88	-										
2. Mother warmth	3.91	0.80	1–5	0.87	0.76 ***	-									
3. Teacher warmth	3.83	0.83	1–5	0.90	0.28 ***	0.32 ***	-								
4. Openness	3.72	0.66	1–5	0.77	0.29 ***	0.29 ***	0.17 ***	-							
5. Positive affect	2.85	0.36	1–4	0.84	0.22 ***	0.26 ***	0.11 **	0.11 **	-						
6. Negative affect	2.17	0.42	1–4	0.77	−0.04	−0.09 *	−0.07	−0.02	−0.49 ***	-					
7. Life satisfaction	3.26	0.45	1–4	0.93	0.58 ***	0.57 ***	0.40 ***	0.34 ***	0.28 ***	−0.07	-				
8. Age	15.91	1.43	13–18	-	0.01	−0.01	−0.01	−0.07	−0.03	−0.07	−0.08	-			
9. Gender ^a^	-	-	1–2	-	0.06	0.05	0.04	−0.13 **	0.17 ***	−0.13 **	0.06	−0.01	-		
10. Socioeconomic status	15.95	1.95	9–25	-	0.10 *	0.09 *	0.01	0.04	−0.04	0.03	0.05	−0.09 *	−0.08	-	
11. Social desirability	5.27	0.82	1–7	0.86	0.44 ***	0.43 ***	0.35 ***	0.41 ***	0.33 ***	−0.16 ***	0.62 ***	−0.04	0.06	0.05	-

*Note*. *N* = 492. ^a^ coded as 1 = boys and 2 = girls. * *p* < 0.05, ** *p* < 0.01, *** *p* < 0.001.

**Table 3:** Model fit indices for different warmth profiles.

	AIC	BIC	aBIC	Entropy	LMR-LRT	BLRT	Smallest Profiles (%)
1-Profile	5593.34	5620.96	5601.91	-	-	-	-
2-Profile	5068.75	5114.79	5083.04	0.80	513.16 ***	532.58 ***	34.2%
3-Profile	4909.64	4974.09	4929.64	0.81	161.02 ***	167.11 ***	8.8%
**4-Profile**	**4840.28**	**4923.15**	**4865.99**	**0.79**	74.54 *	77.36 *	**7.1%**
5-Profile	4732.41	4833.70	4763.84	0.75	115.64 *	120.01 *	2.6%

*Note*. *N* = 738. AIC, BIC, and aBIC are model information criteria, and a lower score indicates a better model fit; entropy refers to the classification accuracy, with a higher score representing better classification accuracy; LMR-LRT and BLRT are two likelihood ratio tests, and a significant *p*-value indicates that a model with *k* + 1 profiles fits significantly better than a model with *k* profiles. Boldface values refer to the optimal solution in this study. * *p* < 0.05, *** *p* < 0.001.

**Table 4:** Regression analysis predicting subjective well-being from warmth profiles, openness to experience, and left-behind status.

Variables	*b*	*b SE*	95% CI for *b*		*t*	*p*
Congruent low	−1.02	0.21	−1.43	−0.61	−4.89	<0.001
Congruent lowest	−1.29	0.29	−1.86	−0.73	−4.47	<0.001
Incongruent moderate	−0.45	0.17	−0.79	−0.12	−2.66	0.01
Openness	0.37	0.13	0.12	0.62	2.93	0.01
Left-behind status ^a^	0.02	0.17	−0.31	0.35	0.10	0.92
Age	0.03	0.04	−0.05	0.12	0.78	0.43
Gender ^b^	0.53	0.13	0.27	0.79	3.98	<0.001
Socioeconomic status	−0.04	0.04	−0.11	0.03	−1.24	0.22
Social desirability	1.01	0.09	0.83	1.20	1.07	<0.001
Congruent low × openness	−0.03	0.28	−0.59	0.52	−0.12	0.90
Congruent lowest × openness	0.46	0.38	−0.29	1.20	1.20	0.23
Incongruent moderate × openness	−0.07	0.24	−0.55	0.41	−0.29	0.77
Congruent low × left-behind status	0.46	0.40	−0.32	1.24	1.15	0.25
Congruent lowest × Left-behind status	1.63	0.55	0.55	2.71	2.95	0.01
Incongruent moderate × left-behind status	0.99	0.33	0.34	1.63	2.97	0.01
Openness × left-behind status	0.90	0.24	0.43	1.36	3.78	<0.001
Congruent low × openness × left-behind status	−0.76	0.57	−1.87	0.35	−1.34	0.18
Congruent lowest × openness × left-behind status	1.88	0.76	0.39	3.38	2.47	0.01
Incongruent moderate × openness × left-behind status	−0.39	0.49	−1.35	0.57	−0.80	0.42

*Note*. *N* = 738. The reference group for warmth profiles was the congruent highest profile. ^a^ coded as 1 = left-behind youth and 0 = non-left-behind youth, ^b^ coded as 1 = boys and 2 = girls.

## Data Availability

The data presented in this study are available on request from the corresponding author.

## Supplementary Materials

The following supporting information can be downloaded at: https://www.mdpi.com/article/10.3390/ijerph19074103/s1, Table S1: The number of participants distributed by eight public schools where the data were collected; Table S2: Mean differences in variables of interest between selected non-left-behind youth and original non-left-behind data pool; Table S3: Mean differences in study indicators across four warmth profiles.
