# Supplementary material for: Openness to Experience Moderates the Association of Warmth Profiles and Subjective Well-Being in Left-Behind and Non-Left-Behind Youth
Source: Int J Environ Res Public Health. 2022 Mar 30;19(7):4103. doi: 10.3390/ijerph19074103 (PMC8998741; doi:10.3390/ijerph19074103)
Supplement: Supplementary file 1 [file ijerph-19-04103-s001.zip › ijerph-1564556-supplementary.pdf]

Supplementary Materials

Table S1. The number of participants distributed by eight public schools where the data were collected

School	Left-behind youth (n = 246)		Randomly selected, non-left-behind youth		Total sample (N = 2671)	
**School A**	79	32.1%	136	27.6%	453	17.0
**School B**	10	4.1%	26	5.3%	162	6.1
**School C**	40	16.3%	75	15.2%	504	18.9
**School D**	27	11.0%	32	6.5%	252	9.4
**School E**	10	4.1%	38	7.7%	218	8.2
**School F**	12	4.9%	29	5.9%	195	7.3
**School G**	37	15.0%	84	17.1%	536	20.1
**School H**	31	12.6%	72	14.6%	351	13.1

Table S2. Mean differences in variables of interest between selected non-left-behind youth and original non-left-behind data pool

Variables	Randomly selected, non-left-behind youth		Total non-left-behind youth		t/χ^2
	M	*SD*	*M*	*SD*	
Father warmth	3.75	0.87	3.69	0.92	1.31
Mother warmth	3.91	0.80	3.89	0.85	0.56
Teacher warmth	3.83	0.83	3.76	0.81	1.55
Openness	3.72	0.66	3.70	0.72	0.43
Positive affect	2.85	0.36	2.84	0.36	0.14
Negative affect	2.17	0.42	2.18	0.42	-0.47
Life satisfaction	3.26	0.45	3.23	0.46	1.24

Age	15.91	1.43	15.57	1.61	4.22
Gender (boys/girls)	221/271	-	832/1101	-	0.56
Socioeconomic status	15.95	1.95	15.68	2.19	2.43
Social desirability	5.27	0.82	5.29	0.83	-0.47

Table S3 Mean differences in study indicators across four warmth profiles

	1. Congruent Low (*n* = 157)		2. Congruent Highest (*n* = 267)		3. Congruent Lowest (*n* = 54)		4. Incongruent Moderate (*n* = 260)				
	M	*SD*	*M*	*SD*	*M*	*SD*	*M*	*SD*	*F*	Partial η^2	Post hoc
Father warmth	2.92	0.53	4.51	0.37	2.13	0.74	3.65	0.57	499.25^{***}	0.67	2 > 4 > 1 > 3

Mother	3.09	0.29	4.68	0.24	2.06	0.43	3.94	0.29	1753.54***	0.87	2 > 4 > 1 > 3
Teacher	3.54	0.77	4.11	0.81	3.28	0.92	3.73	0.72	28.07***	0.10	2 > 4 > 1 > 3

Note. $N = 738$.

*** $p < .001$

Assumption checklist on multiple linear regression

✓ Assumption 1: The relationship between the independent variables and the dependent variable is linear.

Scatterplots of the relationship between each of the independent variables and the dependent variable showed that this assumption had been met. Meanwhile, this assumption could also be detected by bivariate correlations between variables of interest (see Table 1 and Table 2).

- ✓ Assumption 2: There is no multicollinearity in your data.

Analysis of collinearity statistics showed this assumption had been met, as VIF scores on our independent variables were all below 10 (ranged from 1.01 to 8.37).

- ✓ Assumption 3: The values of the residuals are independent.

The Durbin-Watson statistic showed that this assumption had been met, as the obtained value was close to 2 (Durbin-Watson = 1.93, $p = 0.29$).

- ✓ Assumption 4: The variance of the residuals is constant.

The residual plot (see the figure attached below) showed no obvious signs of funnelling, suggesting the assumption of homoscedasticity had been met.

✓ Assumption 5: The values of the residuals are normally distributed.

The Q-Q plot of residuals (see the figure attached below) for the model suggested that the values of the residuals were normally distributed.

✓ Assumption 6: There are no influential cases biasing your model.

Cook's Distance values for the independent variables were all under 1 (ranged from 0.00 to 0.05), suggesting that no significant outliers that potentially biased the model existed.

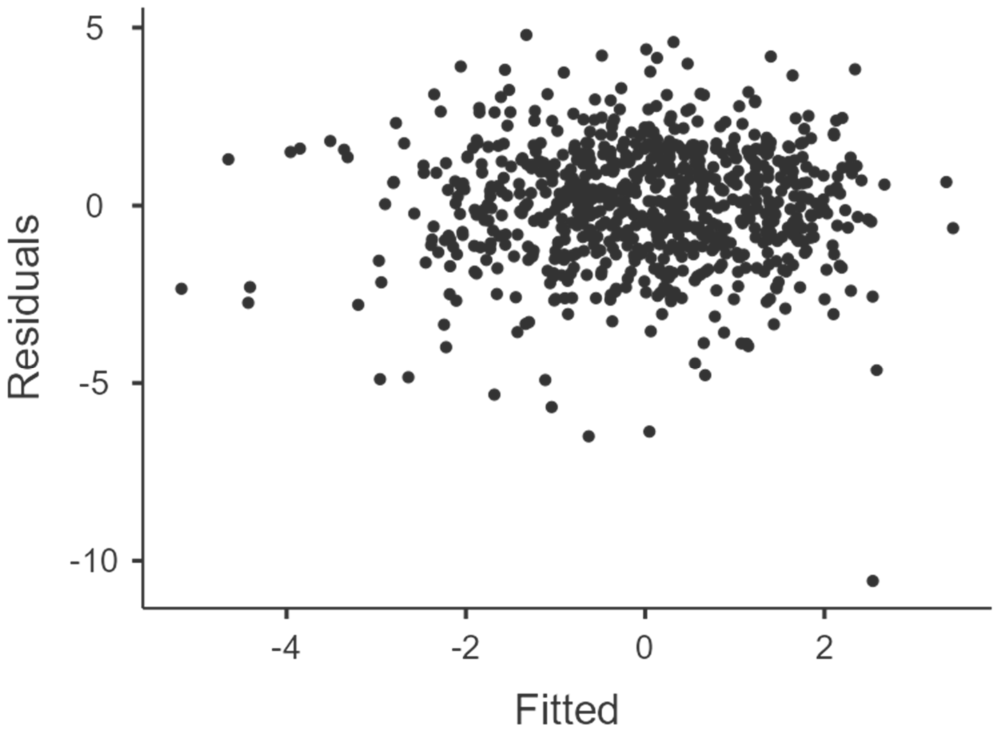
5
0
-5
-10
Residuals
-4
-2
0
2
Fitted

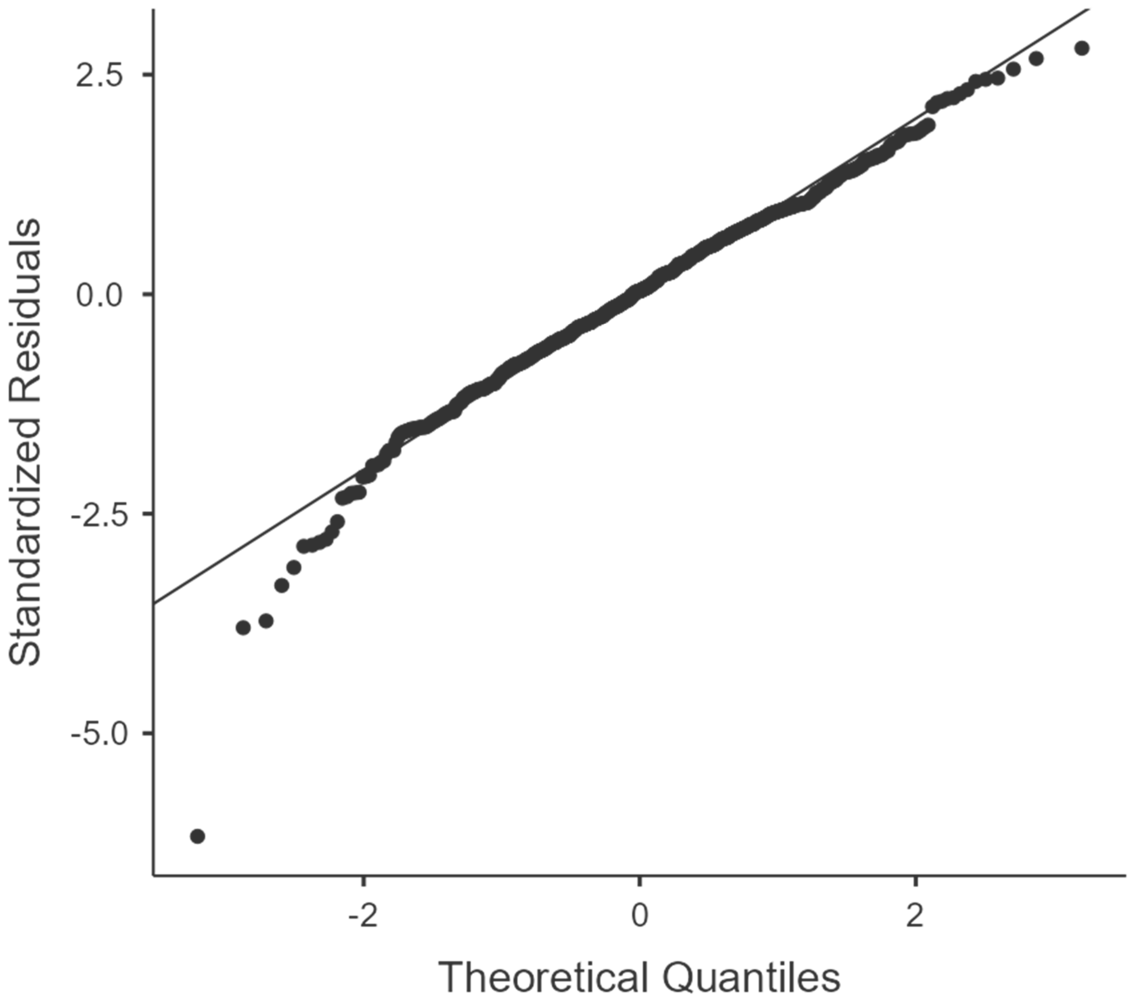

2.5
0.0
-2.5
-5.0
Standardized Residuals
-2
0
2
Theoretical Quantiles
